# Computational Methods in Immunoinformatics: Epitope Discovery and Diagnostic Applications

**DOI:** 10.1021/acsomega.5c05538

**Published:** 2025-09-25

**Authors:** Ana Carolina Silva Bulla, Alessandra Sbano da Silva, Bruno Prado Sereno, Maria Fernanda Ribeiro Dias, Manuela Leal da Silva

**Affiliations:** † Programa de Pós-Graduação em Biologia Computacional e Sistemas, 196605 Instituto Oswaldo Cruz, Fundação Oswaldo Cruz, Avenida Brasil 4365, Manguinhos, Rio de Janeiro, RJ 21040-900, Brazil; ‡ Programa de Pós-Graduação em Biotecnologia, Instituto Nacional de Metrologia, Qualidade e Tecnologia, Av. Nossa Sra. das Graças 50, Xerém, Duque de Caxias, RJ 25250-020, Brazil; § Divisão de Metrologia em Biologia, Diretoria de Metrologia Científica, Industrial e Tecnologia do Inmetro, Instituto Nacional de Metrologia, Qualidade e Tecnologia, INMETRO, Av. Nossa Sra. das Graças 50, Xerém, Duque de Caxias, RJ 25250-020, Brazil; ∥ Centro de Ciências da Saúde - Biomedicina, Universidade Católica de Petrópolis, Rua Benjamin Constant, 213, Centro, Petrópolis, RJ CEP 25610-130, Brazil; ⊥ Programa de Pós-graduação Multicêntrico em Ciências Fisiológicas, Instituto de Biodiversidade e Sustentabilidade NUPEM/UFRJ, Universidade Federal do Rio de Janeiro, Avenida Amaro Reinaldo dos Santos Silva, 764. São José do Barreto, Macaé, RJ 27965-045, Brazil

## Abstract

This review proposes a structured immunoinformatics framework tailored for diagnostic applications, addressing the current gap in standardized pipelines compared to well-established workflows in reverse vaccinology. Immunoinformatics integrates experimental immunology with computational approaches to predict antigen-epitope recognition by B and T cell immune receptors, supporting the identification of diagnostic and therapeutic targets. It enables rapid and cost-efficient prediction of peptide-MHC binding affinity and epitope immunogenicity through machine learning models and specialized algorithms trained on curated immunological data sets. Although epitope prediction pipelines are well-established in vaccinology, standardized frameworks for their application in diagnostic assays remain underdeveloped. This gap reflects challenges in integrating and implementing prediction tools within diagnostic development protocols, which demand distinct validation criteria and clinical applicability compared to vaccine design. We examine key methodological developments, and practical applications are illustrated through case studies involving viral, bacterial, parasitic, and fungal pathogens. Drawing from this assessment, we outline a modular pipeline for epitope prioritization that integrates sequence analysis, structural modeling, consensus-based prediction, and validation strategies. Analysis of the current literature suggests that prediction algorithms utilizing artificial intelligence models yield high accuracy in epitope identification. Following experimental validation, this approach demonstrates considerable potential for implementation in diagnostics. This integrative strategy underscores the value of combining AI-driven prediction, structural modeling, and multiepitope design in translational diagnostics. Epitope-centric approaches promise significant advances in biomarker platforms for diagnostics, vaccine development, and therapeutic design. This review highlights the integrative value of widely adopted immunoinformatics tools and their applicability to serological diagnostics.

## Graphical abstract

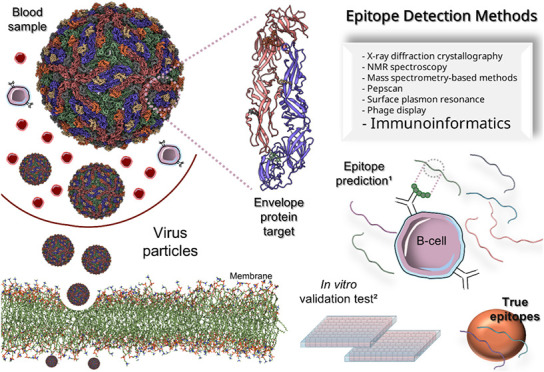

## Introduction

The continuous coevolution of humans and their microbial pathogens has resulted in dynamic host–pathogen interactions that influence disease emergence and progression. These factors have increased antimicrobial resistance profiles and transmissibility, resulting in significant economic and social impacts on populations.^1^ Increasing mutation rates and antimicrobial resistance require the development of more sensitive and specific diagnostic methods, as well as improved prophylactic measures against pathogens. Emerging viral outbreaks with pandemic potential, such as SARS-CoV-2 and highly pathogenic avian influenza (H5N1), represent critical challenges to global public health. Whether emerging (SARS-CoV-2), reemerging (Ebola, Zika), or zoonotic (avian influenza), these outbreaks require diagnostic platforms capable of rapid, specific, and multiplex detection, particularly in epidemic conditions. Accurate diagnoses are essential to guide targeted therapeutic interventions and to inform the development of localized public health strategies.^2–4^

However, several technical limitations, such as low sensitivity, labor intensity, and limited accessibility, continue to hinder the timely and accurate diagnosis of infectious diseases worldwide.^2^ Although etiologic isolation of pathogens can be effective, it is often time-consuming due to technical issues and variability in microscopy.^5^ Molecular diagnostic technologies, such as polymerase chain reaction (PCR), offer higher sensitivity and specificity, except it is limited by high costs, the requirement for advanced laboratory infrastructure, and skilled personnel. Serological assays, including enzyme-linked immunosorbent assays (ELISA) and lateral flow immunoassays, provide rapid and high-throughput results but face problems with cross-reactivity and limited capability to distinguish homologous antigens from related pathogens. These challenges complicate clinical interpretation, particularly in differentiating results related to active infections from immune responses to other diseases.^5,6^

Recent developments in epitope-based diagnostic approaches have shown promise in overcoming these limitations. The use of defined epitopes instead of entire antigens increases the specificity of diagnostic reagents, minimizing cross-reactivity and improving reproducibility. As protein-derived minimal antigenic determinants, epitopes activate both cellular immunity, through recognition of peptide MHC complexes by T cell receptor, and humoral immunity, through binding of the B cell receptor to conformational or linear epitopes.^7–9^ This dual activation highlights the versatility of epitope-based assays, enabling precise detection of immune responses tailored to diverse pathogens and clinical scenarios.^7^

Historically, epitope identification relied exclusively on experimental methods, such as X-ray crystallography, nuclear magnetic resonance (NMR), and hydrogen–deuterium exchange coupled to mass spectrometry.^5,7^ However, the advent of high-throughput genomic sequencing and the increasing availability of epidemiological data have revolutionized immunology research. A pivotal milestone in this evolution was the establishment of the ImMunoGeneTics (IMGT) database by Marie-Paule Lefranc in 1989,^10^ an integrated resource specializing in immunoglobulins (Ig), T cell receptors (TCRs), and major histocompatibility complex (MHC) molecules in vertebrate species. IMGT has been instrumental in advancing computational approaches to epitope prediction and immune system characterization.^11^

Computational immunology, or immunoinformatics, bridges experimental immunology and computer science, enabling the generation of novel hypotheses about immune responses. Integrating curated immunological databases with advanced predictive algorithms facilitates a more comprehensive analysis of the molecular interactions essential for immune function. These databases provide genetic and molecular markers essential to understanding immunogenetics, highlighting the synergy between computational biology and immunology.^11^ The applications of these approaches are illustrated in Figure 1.

**Figure 1:**
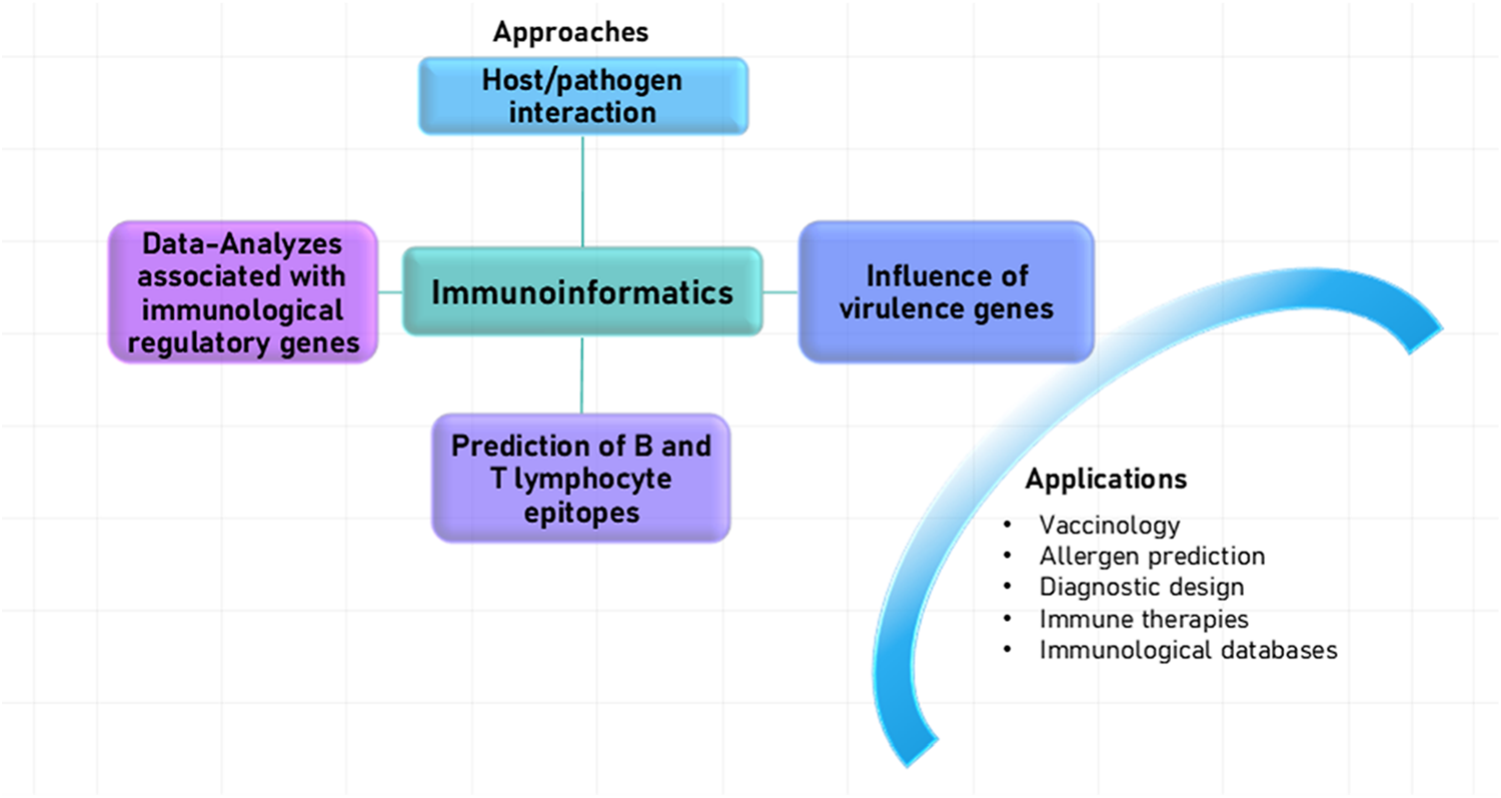
Diagram illustrating different approaches and applications of immunoinformatics. The figure highlights: 1. Computational analysis of host–pathogen interactions, enabling the prediction of immune evasion mechanisms and the identification of molecular targets. 2. *In silico* identification of virulence-associated genes and their influence on immune responses, aiding pathogen characterization and vaccine development. 3. Analysis of immunoregulatory genes to enhance understanding of immune system regulation. 4. Prediction of B and T lymphocyte epitopes using computational strategies to facilitate vaccine development and the implementation of immunotherapeutic applications. These strategies are essential for the advancement of reverse vaccinology, allergen identification, immunotherapy development, and the development of epitope-based diagnostic tools. By enabling the identification of important immune markers, it significantly contributes to the development of more precise diagnostic methods, improving the detection and monitoring of immune-related diseases.^12,13^

The computational strategies and integrated tools described above have proven invaluable for advancing immunological research frameworks. A compelling example of its impact emerged during the COVID-19 pandemic, when computational techniques based on immunoinformatics accelerated the development of vaccines and diagnostic tests.^13^ Despite growing interest, the application of immunoinformatics in diagnostic development remains insufficiently explored in scientific literature.

In contrast to vaccinology, where well-established workflows exist,^14–16^ immunoinformatics-based diagnostic development lacks comprehensive reviews detailing optimized methodologies. This gap limits the refinement of epitope prediction pipelines and the wider adoption of immunoinformatics-based diagnostic approaches for infectious diseases. Addressing this limitation requires a multidimensional understanding of antibody–antigen interactions, incorporating not only the structural features of proteins but also the thermodynamic principles governing these interactions. Reliance solely on static structural information risks oversimplifying the complexity of immunological recognition, potentially leading to biological misinterpretations.^17^

This review provides a comprehensive analysis of *in silico* epitope prediction tools and their applications in diagnostic development, highlighting recent methodological advances. It proposes a structured framework for evaluating these tools and highlights their potential to improve diagnostic accuracy and reliability in diverse infectious disease contexts. Furthermore, this work identifies current challenges and outlines future directions to facilitate the integration of immunoinformatics into routine diagnostic workflows.

## Immunological Aspects

In vertebrates, host defense mechanisms are mediated by two synergistic systems: innate and adaptive immunity. The innate immune system constitutes the first line of defense, mediating rapid and nonspecific responses through physical barriers (skin and mucous membranes), physiological factors (e.g., temperature regulation, acidic pH), cellular components including phagocytic blood monocytes, neutrophils, and tissue-resident macrophages, as well as soluble inflammatory mediators such as serum complement proteins.^12,18^ In contrast, adaptive immunity, which emerged approximately 450 million years ago in jawed vertebrates, confers a highly specific defense mechanism and memory-based protection, and has been the focus of extensive research over the last century.^11,19^

A comprehensive understanding of adaptive immunity at the molecular, cellular, and population levels has been fundamental to advancing vaccine design, diagnostic development, and immunotherapeutic strategies.^20^ Adaptive immunity comprises two main pathways: (i) the cellular immune response, mediated by T lymphocytes, and (ii) the humoral immune response, characterized by the production of antibodies from B lymphocytes.^12,21^ In both pathways, antigen recognition triggers immune activation. An antigen, which can be a protein, carbohydrate, lipid, or another molecule, has specific regions known as epitopes that trigger an immune response.

A single antigenic molecule typically contains multiple epitopes. B cell protein epitopes can be continuous (linear) or discontinuous, whereas T cell epitopes consist of small linear peptides derived from antigenic proteins. B cell receptors (BCRs) and TCRs are responsible for epitope recognition.^22–24^ After differentiation, B cells and antibodies express recognition of soluble antigens or intact proteins by binding to accessible epitopes on the antigen surface (Figure 2). Continuous epitopes consist of contiguous amino acid residues, typically ranging from 5 to 30 residues, while discontinuous epitopes are formed by noncontiguous residues that approach each other through the three-dimensional folding of the protein.^25,26^

**Figure 2:**
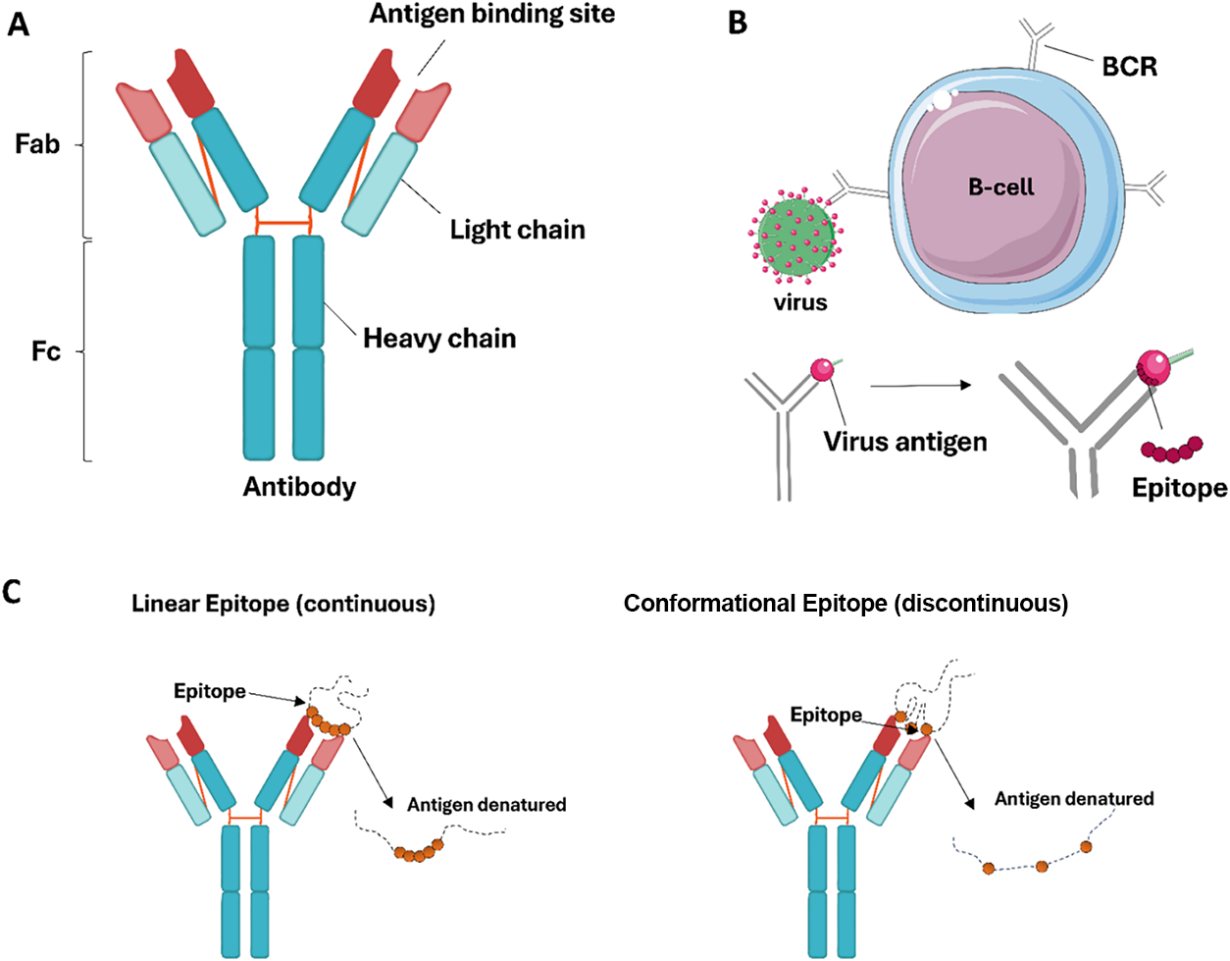
General antibody structure and B cell epitopes. (A) Basic antibody structure. Heavy chains are shown in blue, and light chains are shown in light blue. Antigen recognition regions are shown, and the Fab region is shown in red. Fab: Antigen binding fragment; Fc: Crystallizable fragment. (B) Antigen recognition by B cell receptors (BCR) and antibodies. (C) B cell antigens can be linear or conformational. The regions that constitute the epitope are represented by orange spheres. The antibody, hepatitis virus, and lymphocytes-5 icons from Servier https://smart.servier.com/ are licensed under CC-BY 3.0 (https://creativecommons.org/licenses/by/3.0/); igD icon from DBCLS https://togotv.dbcls.jp/en/pics.html is licensed under CC-BY 4.0 (https://creativecommons.org/licenses/by/4.0/).

Approximately 10% of epitopes are linear (continuous), while most key epitopes are conformational (discontinuous). Identifying these conformational epitopes remains challenging due to their spatial locations depending on the dynamic three-dimensional structure of the antigen, which can vary under physiological conditions. Protein flexibility and conformational changes upon antibody binding further complicate accurate mapping. These complexities necessitate advanced approaches, including structural modeling, to effectively identify conformational epitopes.^26–28^

T cells play a vital role in identifying and controlling pathogens. After antigen degradation and processing, epitopes are presented to T cells as peptides bound to MHC molecules, known in humans as leukocyte antigen (HLA) class I and II. The interaction between the MHC-peptide complex and T cell receptors triggers specific recognition, a critical event that initiates the immune response.^29,30^ This antigen presentation process is essential for orchestrating targeted immune defenses against invading pathogens.

MHC class I molecules are expressed on the surface of nearly all nucleated cells and present endogenous peptides, typically 8–10 amino acids in length, to cytotoxic CD8+ T cell receptors. Peptide presentation by MHC class I allows cytotoxic T cells to recognize and eliminate infected or abnormal cells. In contrast, MHC class II molecules are expressed primarily on antigen-presenting cells, including macrophages, B cells, and dendritic cells, and present longer peptides, derived from exogenous proteins to CD4+ helper T cells.^12,29^ The peptide-binding groove of MHC class II allows the binding of peptides typically 13–25 residues in length (Figure 3).^20^ This ability to bind longer peptides increases the versatility of antigen presentation and the specificity of helper T cell-mediated immune recognition.

**Figure 3:**
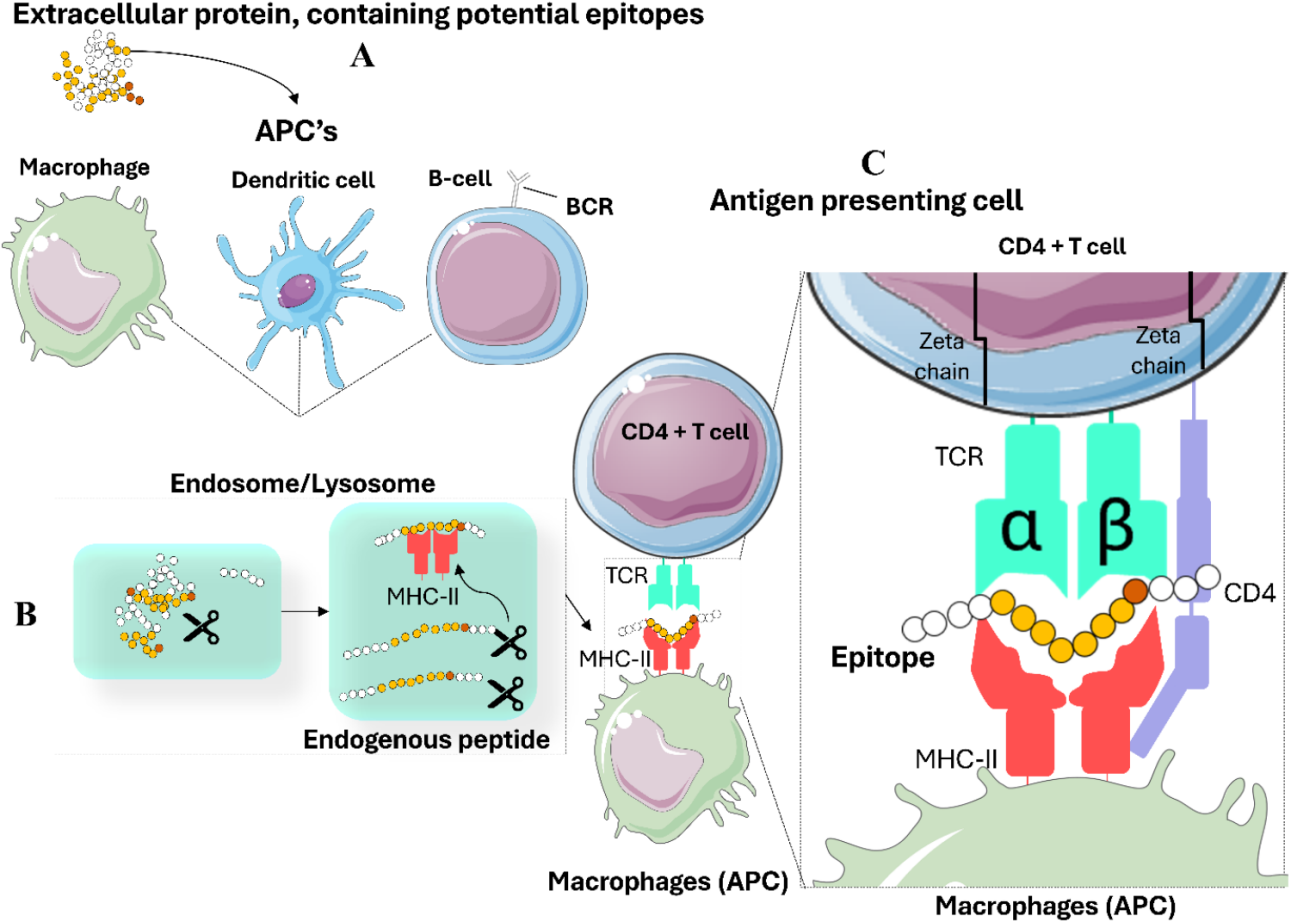
Antigen processing, presentation, and T lymphocyte activation. (A) Antigen capture by Antigen-Presenting Cells (APCs): APCs, including dendritic cells, macrophages, and B lymphocytes, capture extracellular antigens through phagocytosis or receptor-mediated endocytosis. (B) Antigen processing and the MHC II presentation pathway: Extracellular antigens are internalized by APCs into endocytic vesicles (endosomes or phagosomes). These vesicles acidify and fuse with lysosomes, where the antigens are degraded into peptides by proteolytic enzymes. Peptides were then loaded onto Major Histocompatibility Complex class II (MHC II) molecules in specialized vesicles. Peptide-MHC II complexes are transported to the cell surface and presented on CD4 T lymphocytes, initiating an immune response. (C) Recognition by CD4 T lymphocytes and activation of the adaptive immune response: APC presents the processed peptide on MHC II. CD4 T cells recognize the peptide-MHC II complex through their T cell receptor (TCR), which consists of α and β chains. The CD4 coreceptor binds to MHC II to stabilize this interaction. Zeta chains associated with TCR facilitate intracellular signaling, leading to T cell activation. The regions comprising the peptides are represented by the yellow and orange spheres. The icons of APCs and other cells are from Servier https://smart.servier.com/ and are licensed under CC-BY 3.0 (https://creativecommons.org/licenses/by/3.0/).

## Epitope Mapping and Diagnostic Test Development

Serological assays face significant challenges in distinguishing closely related pathogens, often leading to cross-reactivity and complicating accurate diagnosis of infection. Furthermore, in many cases, serological assays lack sufficient sensitivity to accurately monitor disease progression, depending on the pathogen, clinical phase, and immune response dynamics. Efforts to improve serological diagnostics are increasingly focused on improving accuracy and sensitivity. Historically, diagnostic and immunotherapeutic development has relied on the empirical use of whole antigens or on insights derived from pathogen virulence studies. Currently, epitope selection for diagnostic purposes prioritizes those capable of inducing rapid, specific, and measurable immune responses. Consequently, epitope mapping has become essential to identifying antigenic regions with the greatest diagnostic potential.^31–33^

A classic and widely established approach to epitope mapping involves crystallography of the antigen–antibody complex, using X-ray diffraction to resolve its structure. Co-crystallization effectively identifies linear and discontinuous epitopes, a capability not shared by all B cell epitope mapping techniques, making it particularly useful for vaccine design and diagnostic development.^32,34^ Despite its precision, this method faces limitations, as the monoclonal antibodies (mAbs) must be crystallizable and previously characterized, which poses considerable challenges. Moreover, the time investment, technical complexity, and the requirement for large quantities of purified protein-mAb complexes often limit its practical application.^33^ In order to overcome these limitations, alternative strategies for epitope mapping can be employed individually or in combination. Site-directed mutagenesis, a well-established and still widely used technique developed decades ago, supports precise epitope mapping through systematic amino acid substitutions to identify critical contact residues.^35^ Screening of peptide library using phage display enables high-throughput identification of linear and conformational epitopes.^36^ Solution NMR spectroscopy provides residue-level characterization of antibody–antigen interfaces, enabling detailed structural insights.^37^ Mass spectrometry-based methods complement these, enabling high-resolution identification of antigenic segments that interact with antibodies in solution.^38^ Recently, cryo-electron microscopy (cryo-EM) has emerged as a powerful tool for generating efficient and semiquantitative epitope maps in polyclonal antibody responses.^39^ These methods can be applied to the entire proteome of interest or focused on proteins previously identified to induce immune responses. A thorough understanding of the interplay between epitope conformation, surface accessibility, and immune recognition is essential, as effective epitopes must be accessible and correctly oriented to initiate appropriate cellular responses.

The rapid advancement of computational bioinformatics tools applied to immunology is a direct consequence of the exponential growth in genomic, proteomic, and large-scale screening data.^40–42^ Consequently, a new discipline, computational immunology, also known as immunoinformatics, has emerged to analyze and interpret these complex data sets for applied immunological research.

This field is fundamental in disease diagnosis, identification of immunological biomarkers, and providing critical insights into patients’ immune responses. Advanced algorithms and mathematical models are used to reveal complex patterns and interactions between diverse immunological components.^40,43^ The rapid expansion of immunoinformatics tools has revolutionized the development of diagnostics and vaccine candidates, enabling integrated workflows that combine structural, functional, and evolutionary analyses. These tools facilitate the identification of antigenic epitopes recognized by B and T cells, with an emphasis on structural characteristics that modulate immunogenicity.^20,44,45^

Epitope selection and research for diagnostic and vaccine purposes represent distinct but complementary fields within immunology, each guided by specific objectives. Understanding these differences is crucial to optimizing the development of effective immunological tools. Distinct from the pipelines employed in reverse vaccinology, diagnostic approaches must specifically address the risk of nonspecific binding caused by conserved or structurally similar epitopic regions shared between pathogens of the same family, which can result in false positives due to cross-reactivity. High specificity is a fundamental criterion, particularly among closely related pathogen families that exhibit significant sequence and structural similarity. For example, antigenic determinants shared between flaviviruses, such as Dengue, Zika, and West Nile virus, can induce cross-reactivity, leading to false-positive diagnoses and complicating differential diagnosis in endemic regions.^46,47^

Vaccine-targeted epitopes are selected primarily to maximize cross-protection and induce long-lasting immunity. These epitopes are typically derived from conserved immunogenic regions shared across diverse strains and serotypes, ensuring broad reactivity. Such conservation supports the development of effective vaccines against pathogens with high antigenic variability. Additionally, candidate epitopes are prioritized based on their predicted strong binding to multiple MHC alleles and their capacity to induce robust T cell responses. This selection framework is well established and widely known as reverse vaccinology.^47–51^

Historically, diagnostic development has focused predominantly on antibody detection, often neglecting the potential for assessing the cellular immune response.^33,52–54^ However, there is growing recognition of the strategic value of integrating T cell epitope prediction into diagnostic design. While conventional serological tools, such as high-throughput ELISAs for SARS-CoV-2 ^54,55^ and rapid diagnostic tests for Chagas disease, remain valuable for large-scale surveillance, they are primarily designed to capture humoral responses. A prominent work in this context is the study by Ahmed SF, Quadeer AA, Barton JP, and McKay MR (2020), published in PLOS Neglected Tropical Diseases, entitled ‘Cross-serotypically conserved epitope recommendations for a universal T cell-based dengue vaccine.’ Although this study advanced the prediction of conserved T cell epitopes for vaccine purposes, it does not address the integration of cellular immune components into diagnostic assays. Highlighting this aspect in the literature is particularly relevant, as it could enhance both diagnostic sensitivity and specificity. Building on this rationale, the pipeline proposed at the end of this article builds on this rationale, filtering diagnostic epitopes based on antigenicity, structural accessibility, and intrafamily variability to strengthen discriminatory power.^49^

The deliberate incorporation of T cell epitope prediction represents a transformative approach to developing next-generation diagnostics. Modern *in silico* tools for predicting MHC-presented epitopes can achieve accuracy rates above 80–90%,^20,56^ particularly when complemented by structural modeling, enabling the precise identification of peptides capable of triggering antigen-specific T cell responses. This is particularly advantageous in diseases in which cellular immunity plays a central role in pathogenesis, control, or latency.

Several diagnostic advantages arise from this strategy. First, T cell responses often emerge before antibody seroconversion, allowing for more timely detection during the acute phases of infection. Second, T cell-based assays can differentiate between natural infection and vaccine-induced immunity, a distinction essential for epidemiological monitoring and postvaccination assessment.^57^ Furthermore, by targeting multiple epitopes compatible with diverse HLA alleles, it is possible to develop diagnostic tests that maintain high efficacy in genetically heterogeneous populations, thus reducing ethnic bias and improving overall sensitivity.

Moreover, in chronic or latent infections such as HIV and tuberculosis, detection of antigen-specific T cell responses offers a more reliable indicator of disease activity or immune control than serological markers alone.^58–60^ The success of interferon-γ release assays (IGRAs) for tuberculosis, designed using defined T cell epitopes to quantify cellular immunity with high sensitivity, exemplifies the diagnostic potential of this approach

## Epitope Prediction Computational Tools and Databases

### B Cell Epitope Prediction Tools

B cell epitope prediction constitutes a fundamental component of computational immunology. It was the first predictive approach developed based on detailed protein sequence analysis to identify hydrophilic peptide fragments likely to serve as antigenic determinants.^32,44,61^ The seminal work of Hopp and Woods established a correlation between hydrophilic regions and antigenic sites within proteins. These regions are accessible to antibody binding, allowing the characterization of epitopes involved in antigen–antibody interactions. This foundation paved the way for the development of computational epitope prediction as a tool to elucidate immunological recognition mechanisms.^62^

Early computational tools extended epitope prediction by incorporating the physicochemical properties of amino acids.^62,63^ Classification algorithms commonly employ propensity scales to evaluate parameters associated with epitopes, including solvent accessibility,^64^ flexibility,^65^ and location in β-turn regions.^66^ Predictors such as Predictive Estimation of Protein Linear Epitopes (PEOPLE),^67^ PREDITOP,^68^ BcePred,^69^ and the Immune Epitope Database (IEDB) server (http://tools.iedb.org/bcell/)^70^ use these parameters for epitope prediction (Table 1).

**Table 1:** Methods Developed for Discontinuous and Continuous Epitope Prediction^a^

Tools	Link	Dis/Continuous B-Cell	Method	ROC-AUC	Data set composition	refS
BcePred	http://crdd.osdd.net/raghava/bcepred/	Continuous	Physico-chemical propensity scales	58%	Virus, bacteria, protozoa, and fungi.	^69^
Mimox	http://immunet.cn/mimox/	Discontinuous	Mimotope	N/A	Antigens protein, nucleic acids, vertebrates, human, viruses, bacteria, and invertebrates.	^71^
Pepitope (PepSurf)	http://pepitope.tau.ac.il/	Discontinuous	Mimotope	*S: 43%	Antigen proteins, vertebrates, humans, and viruses.	^72^
				*Sp:87%		
Ellipro	http://tools.iedb.org/ellipro/	Both	Structure-based method (geometrical properties)	AUC: 0.73	Virus, bacteria, protozoa, and fungi.	^73^
COBEpro	http://scratch.proteomics.ics.uci.edu/	Continuous	Machine Learning (Support Vector Machine)	AUC: 0.63	MDFT	^74^
BEPro/PEPITO	https://pepito.proteomics.ics.uci.edu/index.html	Discontinuous	Structure-based method (Calculation through scale scores)	AUC: 0.75	Virus, bacteria, protozoa, and fungi.	^75^
Episearch	http://curie.utmb.edu/episearch.html	Discontinuous	Mimotope	*S:43%	Human antigen proteins and HIV.	^76^
				*Sp:90%		
Epitopia	http://epitopia.tau.ac.il/	Both	Machine Learning	AUC: 0.578	MDFT	^77^
EPCES	http://sysbio.unl.edu/EPCES/	Discontinuous	Consensus Score from six different scoring functions	AUC: 0.57	MDFT	^78^
CBTOPE	http://crdd.osdd.net/raghava/cbtope/submit.php	Discontinuous	Machine Learning (Support Vector Machine)	AUC: 0.58	MDFT	^79^
EPSVR	http://sysbio.unl.edu/EPSVR/	Discontinuous	Machine Learning (Support Vector Machine Regression)	AUC: 0.60	MDFT	^80^
EPMeta	http://sysbio.unl.edu/EPMeta/	Discontinuous	Machine Learning (Support Vector Machine Regression)	AUC: 0.638	MDFT	^80^
Bpredictor	http://code.google.com/p/my-project-bpredictor/downloads/list	Discontinuous	Machine Learning (Random Forests)	AUC: 0.598	MDFT	^81^
MIMOPRO	http://informatics.nenu.edu.cn/MimoPro	Discontinuous	Mimotope	*S: 26%	MDFT	^82^
				*Sp: 77%		
Pepmapper	http://informatics.nenu.edu.cn/PepMapper/	Discontinuous	Mimotope	*S: 26%	Antigen proteins, vertebrates, humans and viruses.	^83^
				*Sp: 93%		
DiscoTope 2.0	https://services.healthtech.dtu.dk/services/DiscoTope-2.0/	Discontinuous	Statistics based on log odds ratio	AUC: 0.73	MDFT	^84^
BEST	http://biomine.cs.vcu.edu/datasets/BEST/	Continuous	Machine Learning (Support Vector Machine)	AUC: 0.56	MDFT	^85^
SVMTrip	http://sysbio.unl.edu/SVMTriP/prediction.php	Continuous	Machine Learning (Support Vector Machine)	*S: 80%	MDFT	^86^
				*Sp: 55%		
ABCpred	https://webs.iiitd.edu.in/raghava/abcpred/	Continuous	Artificial Neural Network	AUC: 0.641	MDFT	^87^
EpiPred	https://opig.stats.ox.ac.uk/webapps/sabdab-sabpred/sabpred/more/	Discontinuous	Combination of geometric and antibody–antigen scoring	*S: 44%	MDFT	^88^
				*Sp: n/a		
BepiPred 2.0	http://tools.iedb.org/bcell/	Continuous	Machine Learning (Random Forest)	AUC: 0.62	MDFT	^89^
IBCE-EL	http://thegleelab.org/iBCE-EL/	Continuous	Machine Learning	*S: 63%	MDFT	^90^
				*Sp: 81%		
SEPPA 3.0	http://www.badd-cao.net/seppa3/index.html	Discontinuous	Artificial Neural Network	AUC: 0.71	MDFT	^91^
EpiDOPE	https://github.com/mcollatz/EpiDope	Continuous	Artificial Neural Network (Deep Neural Network)	AUC: 0.67	MDFT	^92^
DLBEpitope	http://ccb1.bmi.ac.cn:81/dlbepitope/	Continuous	Machine Learning (Deep learning)	AUC: 0.73	MDFT	^93^
PECAN	https://github.com/vamships/PECAN	Discontinuous	Machine Learning (Deep learning)	AUC: 0.72	MDFT	^94^
iLBE	http://kurata14.bio.kyutech.ac.jp/iLBE/	Continuous	Machine Learning (Random Forest)	AUC: 0.809	MDFT	^95^
Epitope Vec	https://github.com/hzi-bifo/epitope-prediction	Continuous	Machine Learning (Support Vector Machine)	AUC: 0.778	MDFT	^96^
Epitope 3D	https://biosig.lab.uq.edu.au/epitope3d/prediction	Discontinuous	Machine Learning	AUC: 0.78	MDFT	^97^
NetBCE	https://github.com/bsml320/NetBCE.	Continuous	Machine Learning (Deep learning)	AUC: 0.845	MDFT	^97^
LBCEPred	http://lbcepred.pythonanywhere.com/	Continuous	Machine Learning (Random Forest)	AUC: 0.934	MDFT	^98^
Shuai Lu Predictor	https://github.com/biolushuai/GCNs-and-Att-BLSTM-for-BCEs-prediction	Discontinuous	Machine Learning (Deep learning)	AUC: 0.691	MDFT	^99^
BepiTBR	https://github.com/zzhu33/BepiTBR	Continuous	Machine Learning	AUC: 0.625	MDFT	^100^
CLBTope	https://webs.iiitd.edu.in/raghava/clbtope/#predict	Both	Machine Learning	AUC: 0.800	MDFT	^101^
Epitope1D	https://biosig.lab.uq.edu.au/epitope1d/	Continuous	Machine Learnig	AUC: 0.93	MDFT	^102^
GraphBepi	https://bio-web1.nscc-gz.cn/app/graphbepi	Discontinuous	Artificial Neural Network (Deep Graph Neural Network)	AUC: 0.66	MDFT	^103^
DiscoTope 3.0	https://services.healthtech.dtu.dk/services/DiscoTope-3.0/	Discontinuous	Inverse fold (ESM-IF1) and Positive-Unlabeled Learning	AUC: 0.799	MDFT	^104^
LBCE-BERT	https://github.com/Lfang111/LBCE-BERT	Continuous	Machine Learning	AUC: 0.671	MDFT	^105^
Caliber	https://caliber.math.biu.ac.il/	Both	Machine Learning	AUC: 0.67	MDFT	^106^
SEMA 2.0	https://sema.airi.net/	Discontinuous	Artificial Neural Network (Deep Neural Network)	AUC: 0.76	MDFT	^92^

a The columns represent, respectively: the server’ name; the predictor’s web links, if available; the prediction type (linear, conformational, or both); the underlying algorithm or methods used for the prediction; the area under the curve (AUC) reported in the related articles, or, for predictors that do not calculate ROC-AUC, the sensitivity (S) and specificity (Sp) values are indicated, with an asterisk marking these cases; accuracy-based assessments and recent benchmarking results, if available, along with corresponding citations in the scientific literature; and the type of organism from which the epitopes used for training were derived. For predictors that did not specify the databases in their publications, the acronym Multiple Data set for Training (MDFT) is used. This list includes B-cell epitope prediction tools developed through August 2024.

Subsequently, machine learning approaches such as ABCpred,^107^ COBEpro^74^ and Tri-Peptide Similarity and Propensity scores (SVMTriP) were developed (Table 1). ABCpred, based on Artificial Neural Networks (ANNs), was trained using epitopes from the Bcipep database^108^ and demonstrated a precision of 65.93%. COBEPro applies a Support Vector Machine (SVM) algorithm that integrates secondary structure and solvent accessibility information to calculate epitope propensity scores for individual residues based on peptide fragments within antigen sequences. This method achieved an area under the curve (AUC) of 0.628 for residue-level epitope prediction. SVMTrip combines tripeptide similarity with propensity scores and exhibited a sensitivity of 80.1% and AUC of 0.702 using an IEDB epitope data set. Other machine learning methods, including EpiDope (deep neural network)^92^ showed comparable performance with an AUC of 0.679, whereas LBCEPred^98^ demonstrated superior accuracy and an AUC of 0.934.

BepiPred is among the most widely used B cell epitope predictors. Version 2.0 82 employs a random forest algorithm to predict continuous epitopes. The most recent iteration, BepiPred 3.0,^109^ uses numerical embeddings derived from the ESM-2 protein language model to predict both linear and discontinuous B cell epitopes. When evaluated on a data set of Protein Data Bank (PDB) crystal structures of antigen–antibody complexes, BepiPred 3.0 achieved an AUC of 0.74, outperforming version 2.0, which obtained an AUC of 0.60.

Most B cell epitopes are conformational, and most prediction tools use the three-dimensional structure of proteins as input. Among the first methods developed to predict conformational B cell epitopes are Conformational Epitope Prediction (CEP)^110^ and DiscoTope.^24^ CEP identifies antibody-binding sites on protein antigens through solvent accessibility analysis and demonstrated 75% accuracy on a data set of twenty-one antigen–antibody complexes. DiscoTope 2.0 predicts discontinuous B cell epitopes by combining surface accessibility and amino acid propensity scores, achieving an average AUC of 0.727.

Other structure-based methods, including Epitopia,^77^ ElliPro,^73^ and EpiPred,^111^ have demonstrated promising performance. Epitopia predicts epitopes using protein structural information or amino acid sequences, assigning an antigenic score to each solvent-accessible residue (structure-based) or to all residues (sequence-based). In contrast, ElliPro utilizes geometric properties and accepts both sequence and structural inputs, incorporating homology modeling via Modeler for proteins without resolved structures. Another notable approach, Cbtope, utilizes SVM and reported an accuracy of approximately 86% in epitope prediction.^79^

Mimotope are short synthetic peptides that mimic native epitope structures without sharing identical sequences and have gained increasing interest as antigen surrogates. In this context, mimotope prediction methods, such as Mimox and Pepitope (PepSurf), have also been developed.^71,72^ Mimotopes are peptides identified by their binding affinity to antibodies generated against a native antigen, exhibiting epitope-like properties. Accurate conformational epitope prediction requires both peptide selection based on antibody affinity and detailed three-dimensional structural information on the antigen.

Despite the diverse methods available for B cell epitope identification, accurate prediction of true epitopes remains challenging. This limitation can be addressed through a consensus approach that combines multiple predictors. Integrating the results of three predictors has been shown to significantly reduce false positives.^112^ The evaluation included five prediction tools (BEPro, Discotope, Ellipro, Epitopia, and Seppa) applied to 3D viral structures, with poliovirus and rhinovirus data sets for training and multiple foot-and-mouth disease virus serotypes for testing. As a result, the consensus strategy increased the reliability of epitope identification compared to individual predictors.^112^

However, empirical evaluations show that consensus approaches do not always significantly improve specificity.^113^ For example, while BepiPred and EpiDope generally demonstrate moderate individual performance,^89,92,102,114,115^ ABCpred tends to produce additional false positives due to its higher sensitivity but lower precision, which can negatively affect consensus results.^116^ Moreover, applying stringent criteria that require agreement among all predictors can increase specificity but often results in a substantial loss of sensitivity, risking the exclusion of important epitopes. Therefore, consensus methods should be used cautiously, with careful selection and weighting of individual tools based on their validated predictive profiles and the relevant biological context. These challenges highlight the need for more robust and accurate prediction strategies.

In line with this, a recent study provides a comprehensive evaluation of nine methods for predicting B-cell conformational epitopes, further illustrating the inherent difficulties in this field.^113^ The programs tested included two sequence-based methods (BepiPred-2.0 and CBTOPE), six general structure-based methods (SEPPA3, DiscoTope2, ElliPro, EPSVR, BEpro, and epitope3D), and one antibody-specific structure-based method (EpiPred). These predictors were evaluated using a carefully curated data set of over 250 crystal structures of antibody–antigen complexes, where epitopes were defined based on solvent accessibility changes to minimize redundancy. Despite employing such a robust and comprehensive data set, most predictors performed close to random, achieving an accuracy of approximately 50%. These findings suggest that limitations in predictive performance are not only related to consensus strategies but also stem from the quality of training data and the intrinsic complexity of conformational epitopes.^113^

Furthermore, several benchmark studies have been published using independent data sets derived from databases such as IEDB, PDB, SACS,^117^ AbDb,^118^ UniProt, VDJdb,^119^ and McPAS-TCR.^120^ In order to ensure data set relevance, redundancies must be removed, and additional quality criteria applied, including crystal resolution, antigen size, and surface accessibility.^121–124^

The main discussion of the articles highlighted similar issues, mainly related to sensitivity and specificity, as reported by the predictor developers. Some examples of software tested included ElliPro, BepiPred, Seppa, and DiscoTope, which, like other tools, presented considerably lower AUC values when evaluated on independent data sets, different from those used for training.^121,123^ This discrepancy indicates a clear inflation of results, likely caused by the lack of sufficient experimental data for adequate training, as well as by poor database management. For example, some databases include only positive examples, without nonpositives and decoys samples, which compromises the robustness of the predictive models.^121–124^

With the growing adoption of artificial intelligence, numerous new software programs, especially for local installation, have been developed to improve the accuracy of B cell epitope prediction.^125,126^ Although some studies claim that these tools have achieved state-of-the-art performance,^113^ truly independent benchmarking studies are still scarce, typically focusing on comparing the performance of well-established and widely cited servers.^121–124^

This point exposes several opportunities for advancing the field, such as developing standardized evaluation frameworks based on independent data sets and promoting the crystallization of frameworks without experimental data to expand training resources. Such initiatives would enable the development of truly innovative prediction techniques, helping to navigate the growing number of tools without losing focus on meaningful progress. Importantly, these findings do not suggest disregarding existing prediction tools but rather advocating their cautious and informed use. Some successful cases of the use of prediction tools and *in vitro* methods will be briefly discussed in the following section.

### T Cell Epitope Prediction Tools (MHC II)

Accurate T cell epitope prediction supports a deeper understanding of the interactions between T cell receptors and MHC II molecules, which are essential for adaptive immune responses. Over the years, prediction methods have undergone substantial evolution, propelled by technological advances and a deepening understanding of immunological mechanisms.^21,31,127^ MHC epitope prediction tools, ranging from initial quantitative array techniques to sophisticated computational algorithms, remain fundamental for the characterization of antigen-MHC interactions^128^ (Table 2).

**Table 2:** Methods Developed for T Cell Epitope Prediction (MHC II)^a^

Tools	Link	MHC	ROC-AUC	Method	ref.
SYFPEITHI	http://www.syfpeithi.de/bin/MHCServer.dll/EpitopePrediction.htm	Both	AUC: 0.90	Published motif	^129^
ProPred	http://crdd.osdd.net/raghava/propred/	Class II	AUC: 0.73	Quantitative matrix	^130^
IMTECH	http://crdd.osdd.net/raghava/mhc/	Class II	N/A	Matrix Optimization technique	^130^
RANKPEP	http://imed.med.ucm.es/Tools/rankpep.html	Both	AUC: 0.78	Position Specific Scoring Matrices (PSSMs)	^131^
MHCPred	http://www.ddg-pharmfac.net/mhcpred/MHCPred/	Both	AUC: 0.67	Additive method	^132^
SVMHC	https://www.hsls.pitt.edu/obrc/index.php?page=URL1153429637	Both	N/A	Machine Learning (Support Vector Machine)	^133^
MHC2Pred	http://crdd.osdd.net/raghava/mhc2pred/	Class II	AUC: 0.36	Machine Learning (Support Vector Machine)	^134^
NNAlign 2.0	https://services.healthtech.dtu.dk/services/NNAlign-2.0/	Both	N/A	Artificial Neural Network	^135^
NetMHCpan 4.0	https://services.healthtech.dtu.dk/services/NetMHCpan-4.0/	Both	AUC: 0.90	Artificial Neural Network	^136^
NetMHCII 2.3	https://services.healthtech.dtu.dk/services/NetMHCII-2.3/	Class II	AUC: 0.85	Artificial Neural Network	^127^
TCRMatch	http://tools.iedb.org/tcrmatch/	Both	AUC: 0.68	Machine Learning	^137^
Deepitope	Unavailable	Class II	AUC: 0.85	Artificial Neural Network (Convolutional Neural Network)	^138^
CAPTAn	https://gitlab.com/xavier-lab-computation/public/captan	Class II	AUC: 0.83	Machine Learning (Deep Learning)	^139^
TRAP	https://github.com/ChloeHJ/TRAP	Class II	AUC: 0.71	Machine Learning (Deep Learning)	^140^
TCRDock	https://github.com/phbradley/TCRdock	Both	AUC: 0.85	Machine Learning (Deep Learning)	^141^
MixMHC2pred 2.0	https://github.com/GfellerLab/MixMHC2pred	Class II	AUC: 0.85	Machine Learning	^142^
Prime 2.0	http://ec2-18-188-210-66.us-east-2.compute.amazonaws.com:3000/	Class II	AUC: 0.85	Machine Learning	^143^
TROLLOPE	https://pmlabqsar.pythonanywhere.com/TROLLOPE	Both	AUC: 0.76	Machine Learning	^144^
GTE	https://github.com/uta-smile/GTE	Both	AUC: 0.75	Artificial Neural Network	^145^
HLAIIMaster	https://github.com/TomasYang001/HLAIImaster	Class II	AUC: 0.93	Machine Learning (Deep Learning)	^146^

a The columns represent, respectively: the server name and its variations; the web link to access the predictor, if available; the type of prediction performed (MHC II only or MHC I and MHC II); the algorithms and methods used for the prediction; the area under the curve (AUC) reported in the related articles; and accuracy-based evaluations and recent benchmarking results, if available, along with corresponding citations in the scientific literature. This list includes T-cell epitope prediction tools developed through August 2024.

The first MHC class II prediction methods, developed in the 1990s, relied on conserved motifs identified in known immunogenic peptides. These algorithms rely on conserved motifs and position-specific scoring schemes, based on experimentally derived amino acid preferences, to predict MHC binding. Tools such as SYFPEITHI^129^ and EPIPREDICT^147^ were among the first to apply this strategy, offering MHC class II binding predictions based on previously characterized motifs. Notably, SYFPEITHI also serves as a comprehensive MHC epitope database, providing detailed information on peptide sequences recognized by diverse MHC alleles. This resource has been instrumental in developing predictive models and advancing our understanding of antigen-MHC interaction mechanisms.

With the advancement of bioinformatics, methods have emerged that use Position-Specific Scoring Matrices (PSSMs) to assign position-specific weights to peptides, aiming to increase prediction accuracy. Outstanding tools using this approach include ProPred,^130^ IMTECH,^148^ and RANKPEP,^131^ which have contributed significantly to the improved identification of immunogenic epitopes, particularly for MHC class II molecules.

In the early 2000s, sophisticated statistical models, such as MHCPred^132^ were introduced, employing an additive method to calculate peptide affinities for MHC, thereby producing more robust predictions than previous approaches. The subsequent development of classical machine learning techniques, particularly SVM, further improved epitope identification. Tools such as SVMHC,^133^ MHC2Pred,^134^ and TCRMatch^137^ applied these methodologies to refine prediction accuracy and improve the reliability of results.

A fundamental approach in immunoinformatics involves incorporating immunological data from experimental animals, exemplified by tools such as MHC2Pred. This strategy is valuable for investigating genetic variability between species, facilitating informed decision-making in preclinical animal studies, and contributing to the reduction of animal use in research.

Important advances in this field include tools such as NNAlign 2.0,^127,149^ NetMHCpan,^150^ and NetMHCII.^149^ These tools highlight the critical ability to incorporate MHC genetic diversity, which is a principal factor in epitope prediction. For instance, NetMHCpan accounts for heterogeneity in the human population, revealing variations in antigen-MHC binding across different ethnic groups. These cutting-edge methods leverage extensive training data sets and ANNs to obtain high-accuracy predictions of MHC class II epitopes. By integrating quantitative binding data with mass spectrometry-derived ligand profiles, ANNs have substantially enhanced T cell epitope prediction, enabling the analysis of large experimental data sets and the identification of complex MHC binding patterns across diverse MHC II alleles.

The NetMHCII server predicts the binding of peptide epitopes to specific MHC class II alleles, supporting the design of targeted immunotherapy. In contrast, NetMHCpan uses a pan-specific algorithm to analyze a wide range of MHC II alleles, addressing genetic heterogeneity between populations. Both NetMHCII and NetMHCpan utilize large experimental data sets and artificial neural networks to enhance prediction accuracy. For example, NetMHCII was used to identify conserved epitopes across dengue virus serotypes, while NetMHCpan guided the design of a neonatal rotavirus vaccine.^151,152^

The predictive performance of these tools has improved significantly through continuous updates. NetMHCpan 4.1 integrates an expanded data set and refined machine learning algorithms, enabling more accurate predictions across a wider range of MHC alleles. This version utilizes custom machine learning strategies to combine different types of training data, achieving a state-of-the-art that surpasses previous models. Similarly, NetMHCII^153^ was updated to version 3.2, incorporating a comprehensive data set of MHC class II binding interactions and enhanced predictive models to improve epitope identification. MHCPred advanced to version 2.0 in 2006, featuring improved algorithms and an expanded peptide-MHC interaction database, although it has not been updated since.^154^

VacPred and MHCBN^148,155^ are noteworthy tools contributing to vaccine development and immunoinformatics research. VacPred facilitates rational vaccine design by identifying T cell antigens in bacterial proteins, targeting specific regions to induce robust and specific immune responses necessary for effective immunization. MHCBN advances the understanding of complex antigen-MHC interactions through the analysis of large data sets. One practical application involved the use of VacPred and NetMHC tools to design and validate a peptide-based vaccine against canine circovirus, demonstrating stable epitope-receptor interactions and CD4+ T cell-dependent antibody production.^151^

## Additional Methods and Tools

In order to increase the diagnostic accuracy and specificity of epitope-based predictions, a variety of databases, computational strategies, and complementary tools are employed outside the pipeline. These tools contribute to the refinement of epitope selection by integrating insights into antigenicity, immunogenicity, and cross-reactivity derived from molecular, genomic, and immunological layers.

The IEDB^70^ is a rigorous resource dedicated to infectious diseases, allergies, and immunity, offering comprehensive predictive tools for epitope analysis. Developed by La Jolla Institute of Allergy and Immunology (LIAI) with support from the National Institute of Allergy and Infectious Diseases (NIAID) and the National Institutes of Health (NIH), the IEDB compiles experimental data that identify and characterize epitopes, its epitope-specific immune receptors, and associated details, including host organisms, immunological exposures, and elicited immune responses. The platform is continuously enhanced with new epitope prediction and analysis tools, supporting the advancement of epitope-based therapies.^19,70^

VaxiJen^156^ is the first alignment-independent antigen prediction server, classifying antigens solely based on the physicochemical properties of proteins, without the necessity of sequence alignment. Users submit protein sequences individually or as multiple sequence files in FASTA format. Antigenicity is evaluated against a predetermined threshold derived from the server’s training set. The output provides the target name, protein sequence, prediction probability, and classification as protective antigen or nonantigen, based on a predefined threshold.

Certain protein regions lack well-defined tertiary structures under native conditions and are classified as intrinsically disordered proteins (IDPs) or intrinsically disordered regions (IDRs). These regions exhibit high conformational flexibility and have been associated with antigenicity. A seminal paper published in 1984 first demonstrated this property of antigenic determinants,^157^ and more recent research in 2018 reinforced the correlation between IDRs and epitopes used in vaccine development in various organisms.^158^ The Intrinsically Disordered Prediction (IUPRED) tool has since facilitated the identification of such regions, supporting epitope selection. Additionally, web-based tools such as Epitope Cluster Analysis (http://tools.iedb.org/cluster/help/), Epitome (https://www.rostlab.org/services/epitome/), and ProtParam (http://web.expasy.org/protparam/) assist in identifying antigenic residues and evaluating the physicochemical properties of predicted epitopes.

Pangenomic analysis provides an evolutionary perspective by identifying antigenic regions conserved across multiple strains or species. This strategy increases the robustness of diagnostic targets by prioritizing epitopes encoded in the core genome, thereby reducing false negatives and increasing applicability across diverse isolates. This approach is particularly useful for pathogens with high genetic variability, such as *Mycobacterium tuberculosis*, *Klebsiella pneumoniae*, and *Streptococcus agalactiae*, where conserved epitopes ensure more consistent diagnostic performance.^159–161^

After candidate epitope selection, molecular docking serves as a critical computational tool for evaluating its interactions with specific MHC alleles or diagnostic antibodies, offering insights into conformational stability, binding energy, and interface complementarity .^162^ In addition, continuous improvements in docking algorithms and scoring functions have made the method more robust and accessible, expanding its applicability beyond drug discovery to include fields such as natural product research and food science. Widely adopted tools, such as HADDOCK^163^ and ClusPro,^164,165^ are routinely used to simulate these interactions. HADDOCK, for example, has demonstrated flexibility in modeling complex biomolecular interactions, including protein–glycan systems ClusPro has consistently ranked among the top performers in CAPRI evaluation rounds, validating its accuracy in predicting protein–protein complexes. The combined or iterative use of these platforms further optimizes *in silico* screening and prediction of biomolecular complexes.^165^

## Epitope Prediction and Diagnosis Application

Epitope identification is highly dependent on the research context, requiring careful consideration of factors including pathogen, source protein, and host organism when designing predictive workflows.^166,167^ To address these challenges, researchers employ *in silico* and *in vitro* methods, each offering complementary insights into epitope characterization and validation. This section reviews key studies in this area, aiming to highlight optimal peptide selection criteria and propose strategies to improve workflows for the development of diagnostic tools based on epitope-antibody recognition.

An accurate diagnosis of Hepatitis C Virus (HCV) infection is crucial for effective disease management, particularly due to the virus’s ability to evade immune responses. A comprehensive study on neutralizing epitopes in the HCV E2 envelope glycoprotein used computational methods to predict epitope accessibility and structural conformation.^168^ Their bioinformatics-driven approach identified three major overlapping neutralizing sites with high immunogenic potential: antigenic site 412 (AS412), antigenic site 434 (AS434), and antigenic region 3 (AR3).

The effectiveness of their computational framework was demonstrated by its ability to guide precise epitope selection, subsequently validated through ELISA assays and structural analysis of epitope-antibody complexes. Similarly, bioinformatics tools were employed to identify epitopes to investigate SARS-CoV-2 135. Linear epitopes were mapped to four viral proteins: S (spike), nsp3, N (nucleoprotein), and nsp12, with over 80% comprising fewer than 15 amino acids. Conservation analysis revealed that the most conserved epitopes resided in critical enzymes, such as nsp12. Conformational epitopes were predicted using molecular structures derived from experimental data and high-quality homology models, supported by DiscoTope2. These analyses highlighted flexible protein regions potentially involved in modulating immune responses. The study identified the nucleoprotein RNA-binding domain (N-Nt), the nsp3 protease, and the nsp16 methyltransferase as key candidates for diagnostic applications. Experimental validation using ELISA testing with patient samples confirmed that both N-Nt and the spike receptor-binding domain (S-RBD) exhibited significant diagnostic potential, demonstrating high sensitivity and specificity.

In contrast, SARS-CoV-2 B cell epitopes were identified only by *in silico* methods, without subsequent experimental confirmation.^169^ They analyzed the structural and immunological properties of the S and N proteins using computational tools. Epitope mapping identified five antigenic sites on the S protein and three on the N protein, employing IEDB, BepiPred, Emini Surface Accessibility, and the Kolaskar and Tongaonkar antigenicity scale. Based on these predictions, multiepitope constructs, designed sequences that bind multiple immunodominant epitopes on a single peptide or protein, were designed to enable broader antibody recognition and enhance diagnostic performance.^170^ Its physicochemical properties were evaluated using ProtParam and SOLpro, and codon optimization for *Escherichia coli* expression was performed with J-CAT. Structural validation confirmed the integrity and immunogenic potential of the peptide using Ramachandran plots and ProSA-web. The absence of experimental validation limits the immediate applicability of these findings.

A comprehensive immunoinformatic pipeline was employed to investigate the immune response against Epstein–Barr virus (EBV) in patients with systemic lupus erythematosus, aiming to identify potential epitopes and better understand the mechanisms underlying disease progression.^171^ Secondary structure prediction of EBV proteins EA, MA, LMP-1, and LMP-2A was performed with the Protean module in DNAStar, selecting Amphiphilicity (Eisenberg), Secondary structure (Garnier-Robson and Chou-Fasman). TransmembrVane domains were identified using TMpred in the EXPASY server. Surface properties, including surface probability (Emini), flexibility (Karplus-Schulz), hydropathy (Kyte-Doolittle), and antigenicity (Jameson-Wolf), were analyzed using DNAStar modules, while polarity was assessed with ProtScale (Polarity/Zimmerman scale) on EXPASY. Based on these analyses, B-cell epitopes with high antigenic potential and physicochemical similarity to lupus-related autoantigens were identified. The selected epitopes were synthesized and validated through *in vivo* immunization in mice, confirming their immunogenicity through serum analysis.

The value of combining *in silico* and *in vitro* methods was demonstrated by a study that investigated serologic differentiation between Dengue and Zika viruses.^172^ They predicted 24 linear B cell epitopes and refined their selection through structural and antigenicity analyses. Validation with sera from monoinfected rabbits revealed distinct immunogenic profiles for each virus, identifying epitopes with strong discriminatory potential for serodiagnostic applications.

Tuberculosis (*Mycobacterium tuberculosis*) remains an important bacterial pathogen and a leading cause of death worldwide, particularly affecting immunocompromised individuals. Effective disease control depends on early diagnosis, appropriate treatment, and vaccination. In this context, a study identified four potential antigens (HspX, EspC, CFP7, and PPE57) for serodiagnostic development. After identifying the B cell epitope, fusion proteins were designed and demonstrated enhanced sensitivity in ELISA assays using serum from tuberculosis patients.

Other relevant bacterial pathogens include *Mycoplasma hominis* and *Rickettsia rickettsii*. *M. hominis*, an opportunistic bacterium of the genital tract, is associated with conditions such as vaginosis, pelvic inflammatory disease, and infective endocarditis. Traditional culture methods remain the diagnostic gold standard; however, bioinformatics-driven prediction of linear B cell epitopes from the membrane proteins P120 and P80 has enabled the development of a chimeric antigen, recombinant proteins composed of antigenic regions derived from different sources, which have demonstrated immunoreactivity in ELISA assays.^173^ Additionally, four epitopes from the OmpA protein in *R. rickettsii*, the causative agent of Rocky Mountain spotted fever, were identified via ELISA using opossum sera, achieving an AUC of 0.886 and sensitivity and specificity of 85.7 and 100%, respectively, highlighting a promising diagnostic potential.^174^

Moreover, human schistosomiasis is a neglected tropical disease with a significant public health impact, affecting a large global population. The diagnostic potential of *Schistosoma japonicum* (SjEV) extracellular vesicle (EV) proteins was evaluated, with several SjEV proteins analyzed for B cell epitope prediction using DNASTAR software, and selected peptides assessed for diagnostic purposes via ELISA.^175^

Another neglected tropical disease, cysticercosis, caused by *Taenia solium*, affects multiple organs or tissues, with neurocysticercosis being the most prevalent parasitic infection of the human central nervous system.^176^ The synthetic mimotope peptide NC41 demonstrated high diagnostic performance, achieving sensitivity and specificity of 97.5% and an AUC of 0.997. Given the conserved nature of PEPCK, computational modeling and analysis (SWISS-MODEL, Pepitope server) of PEPCK from *Echinococcus vogeli* and *E. granulosus* revealed important structural differences in the binding region of NC41 compared to that of *T. solium*. This differentiation explains the absence of cross-reactivity with sera from Echinococcus patients, highlighting the specificity of NC41 for neurocysticercosis diagnosis.^177^

In protozoan parasites, considerable research has focused on identifying diagnostic epitopes. In particular, linear B cell epitopes within the *Leishmania infantum* proteome have been predicted.^178^ To ensure specificity and diagnostic accuracy, candidate antigens were prioritized based on expression during vertebrate host stages, high antigenicity, and ability to discriminate *L. infantum* from closely related pathogens, such as *Trypanosoma cruzi*. Epitope prediction was performed using the BepiPred software, complemented by structural accessibility assessment with IUPred to evaluate epitope availability for antibody binding.

After epitope identification, six peptides were prioritized and synthesized for ELISA validation in sera from patients with visceral leishmaniasis and control subjects. The peptide cocktail (Mix IV: 3 + 6) demonstrated excellent diagnostic performance, achieving 100% accuracy and a kappa coefficient of 1.000, effectively distinguishing *L. infantum* and *T. cruzi* infections. Statistical analyses, including ROC curves, were used to optimize cutoff values and reinforce result reliability. These results reinforce the effectiveness of computational approaches in antigen discovery and their potential to enhance diagnostic precision.

Despite these promising findings, broader clinical validation is necessary to confirm diagnostic efficacy in diverse populations. This concern is particularly relevant due to the genetic variability among geographically distinct *T. cruzi* isolates, which can impact antigenic profiles and compromise test performance. A study emphasized this variability and identified polymorphic and conserved sequences capable of discriminating between parasite strains.^106^ Validation of a conserved peptide via ELISA demonstrated 95.8% sensitivity and 88.5% specificity, indicating substantial potential for enhancing the diagnosis of Chagas disease.

Furthermore, the GRA4 protein of *Toxoplasma gondii*, the causative agent of toxoplasmosis, was investigated.^179^ Using DNASTAR PROTEAN software, they assessed the hydrophobicity, accessibility, and antigenicity of amino acids to identify epitopes with high antigenic potential. Eleven peptides were synthesized and evaluated by ELISA with sera from pigs infected with the Gansu Jingtai isolate of *T. gondii*. Three peptides were consistently recognized by all sera, indicating promising candidates for the diagnosis of porcine toxoplasmosis. However, the study observed variability in the immune response of pigs during infection, highlighting the need for further evaluation of epitope stability over time to ensure diagnostic reliability.

A diagnostic kit based on a multiantigen approach incorporating GRA7, SAG1, and ROP1 was developed using *in silico* strategies to improve the diagnosis of toxoplasmosis.^180^ Multiple epitope prediction tools, including ABCpred, IEDB, and DiscoTope, were used to identify linear and conformational epitopes. After structural modeling using I-TASSER, the predicted epitopes were validated and cloned into *Escherichia coli* BL21 (DE3) for recombinant protein expression. The resulting recombinant multiepitope protein (rMEP), approximately 24 kDa in size, exhibited strong reactivity with *T. gondii*-positive sera, indicating that the combination of three antigens can increase diagnostic specificity and sensitivity. Nonetheless, further validation is needed to confirm performance in diverse human populations from endemic regions. Additionally, the lack of comparison with commercial diagnostic kits limits the assessment of its clinical utility.

The importance of genetic conservation in biomarker selection has also been highlighted, exemplified by the HDP protein of *Plasmodium falciparum*, whose low genetic variability renders it a promising candidate for diagnostic applications.^181^ This aligns with the concept of immunological stability, which is particularly crucial when considering temporal fluctuations in immune responses during infection. Expanding on this notion, a recombinant multiepitope antigen was designed to optimize immunodiagnostic test performance.^179,180^ Notably, this multiepitope construct enhances test reliability by maximizing reactivity with specific antibodies while excluding immunologically irrelevant regions, representing a fundamental advance in the development of diagnostic assays.

Finally, human fungal infections, especially those caused by *Histoplasma capsulatum*, deserve significant attention due to their high morbidity and mortality in immuno-compromised patients. Traditionally, the diagnosis of histoplasmosis has been based on antigen detection from *H. capsulatum* culture extracts. Novel antigenic targets involved in host–pathogen interactions have been identified for serodiagnostic use.^182^ Using the prediction of B cell epitopes in immunogenic proteins recognized by antibodies from patient sera, they identified 14 epitopes unique to *H.* *capsulatum*, offering the potential for greater diagnostic specificity.

The studies discussed above highlight the need for a robust framework to experimentally validate epitopes and ensure their effectiveness in terms of accuracy and specificity. A summary of the methods applied is presented in Table 3, where quantitative data (such as AUC, sensitivity, and specificity) and qualitative data (such as SDS-PAGE gel results) are provided by the authors. The integration of computational methods with rigorous laboratory validation steps is crucial to advancing the clinical applicability of epitope-based diagnostics and immunotherapies. Therefore, the validation process must include:

- Validation in diverse populations and clinical settings: multicenter studies are essential to ensure diagnostic reliability across different genetic and immunological profiles. For tuberculosis, epitopes should be evaluated in both active TB patients and BCG-vaccinated individuals to minimize confounding factors.
- Animal models for evaluating immune response: vaccine candidates must be evaluated for CD4+ and CD8+ T cell activation, cytokine production, and induction of immune memory. Promising epitopes identified in Leishmania spp., *Schistosoma mansoni*, and *Mycobacterium tuberculosis* require in vivo validation to confirm immunogenicity and safety before progressing to clinical trials.
- Nanotechnology and biomaterial applications: epitope-functionalized nanoparticles can improve diagnostic stability and sensitivity. Biosensor-based epitope immobilization may enable earlier and more accurate detection in toxoplasmosis and neurocysticercosis, promoting point-of-care diagnostics.

**Table 3:** Elements and Performance Metrics in Reviewed Diagnostic Marker Studies: A Summary of Methods and Outcomes^a^

	*In silico test*		*In vitro test*								
							Sample				
Pathogen	Epitope prediction tools	Additional tools	Test	N° Peptide synthesized	N° True epitope	Epitope name	Origin	N° Positive/Negative	Metric	Value	Reference
VIRUS	Kolaskar and Tongaonkar antigenicity from IEDB	-	ELISA	4	4	1A	Human	29/25	Optical Density	0.28 ± 0.14 (*p: 0.007)*	^183^
						4D				0.28 ± 0.14 (*p:* 0.038)	
						1B				0.29 ± 0.12 (*p*: 0.002)	
						Consensus				0.55 ± 0.19 (*p*: 0.033)	
	BepiPred2, DiscoTope2	Robetta, PrQ3DEMA	ELISA	Epitopes predicted from five expressed proteins	Two candidate proteins	N-terminal fragment of the nucleoprotein	Human	78/22	Sensitivity/Specificity	87%/100%	^184^
						Receptor-binding domain of the spike protein				92%/100%	
	Amphiphilicity-Eisenberg, Secondary structure-Garnier-Robson, Secondary structure-Chou-Fasman, Surface probability-Emini, Flexibility–Karplus-Schulz, Hydropathy–Kyte-Doolittle, and Antigenicity–Jameson-Wolf from DNAStar	TMpred from EXPASY	ELISA	10	6	EP1	Mice	5/5	AUC/Sensitivity/Specificity	0.655/38.7%/95.3%	^171^
						EP2				0.618/27.7%/98.4%	
						EP3				0.655/42%/96.9%	
						EP4				0.878/71.4%/93.7%	
						EP6				0.749/62.2%/82.8%	
						EP10				AUC: 0.590/88.2%/31.2%	
	Bepipred1.0	-	ELISA	24	3	Pep1	Human	20/20	AUC/Sensitivity/Specificity	AUC: 0.9088/59.46%/95%	^172^
						Pep2				AUC: 0.8278/45.07%/90.91%	
						Pep3				AUC: 46.81/31.51%/100%	
BACTERIA	Bepipred1.0, Bepipred-2.0	Expasy, RaptorX, ModRefiner, MolProbity, PROCHECK, CPORT	ELISA	Epitopes predicted from three expressed proteins and three expressed fusion proteins	A fusion protein candidate****	HspX-EspC-CFP7-PPE57****	Human	230/110	AUC/Sensitivity/Specificity	AUC: 0.9440/69%/99%	^185^
	Kolaskar & Tongaonkar Antigenicity, Karplus & Schulz Flexibility Prediction, and Parker Hydrophilicity Prediction from IEDB	Swiss-Model, Phyre2, ExPASy	ELISA	A chimeric protein candidate (fusion of 10 peptides)	A chimeric protein candidate	Mh128	Human	15/15	Optical Density	2.73 ± 0.15	^173^
	Bepipred-1.0, Epitopia	-	ELISA	1	1	OmpA-pLMC	Capybara	16/12	Optical Density	2.389 ± 0.761 - *P: 0.048*	^174^
							Horse	9/12		0.456 ± 0.411 - *p* = 0.67	
							Opossum	14/5		0.720 ± 0.440 - *P: 0.027*	
PROTOZOAN	Bepipred1.0	IUPred	ELISA	6	3	Peptide-6	Human	70/138	AUC/Sensitivity/Specificity	0.999/100%/99.28%	^178^
						Mix III (peptides 2 + 6)				0.994/100%/99.28%	
						Mix I (peptides 2 + 3+6)				0.999/100%/97.83%	
	Bepipred1.0		Immunoblotting, ELISA	150	36 reactive (mouse serum), 4 tested (human serum), 3 genotype reactive	C6_30_cons	Human (*T. cruzi* genotype TcI or TcII)	16/24	Sensitivity/Specificity/Accuracy	95.8%/88.5%/92.7%	^106^
						A6_30_col				100% /91.9%/92.6%	
						B2_30_y				80%/94.8%/92.6%	
	Garnier-Robson and Chou-Fasman, Kyte-Doolittle, Karplus-Schultz, Emini algorithm, and the Jameson-Wolf from DNAStar	-	ELISA	11	3	P2	Pig	51/10	Optical Density	NQ	^179^
						P10				NQ	
						P11				NQ	
	Chou and Fasman β-turn, Emini Surface accessibility, Karplus and Schulz flexibility, and Kolaskar and Tongaonkar antigenicity from IEDB. Bepipred-1.0, ABCpred, Lbtope, CBTOPE, DiscoTope 2.0	I-TASSER	Western blot immunoassay	A multiepitope (eight epitopes)	A multiepitope (eight epitopes)	rMEP	Human	10/NA	SDS-PAGE gel	NQ	^180^
	Bepipred2.0	-	ELISA	2	2	Peptide 1	Human	122/NA	Optical Density	Peptide 2 elicited a significantly stronger IgG response than peptide 1 (*P* < 0.0001).	^181^
						Peptide 2					
WORM	Chou–Fasman, Jameson–Wolf, Kyte–Doolittle, Emini algorithm, and Karplus–Schultz from DNAStar	-	ELISA	10	2	Peptide 68	Mice	13/NQ	Optical Density	*P* ≤ 0.001 (Positive × Control)177	^175^
							Rabbit	13/NQ		*P* ≤ 0.001 (Positive × Control)	
							Human	10/NQ		*P* ≤ 0.001 (Positive × Control)	
						Peptide 71	Mice	13/NQ		*P* ≤ 0.001 (Positive × Control)	
							Rabbit	13/NQ		*P* ≤ 0.001 (Positive × Control)	
							Human	10/NQ		*P* ≤ 0.001 (Positive × Control)	

a IEDB: Immune Epitope Database; ELISA: Enzyme-linked Immunosorbent Assay; SDS-PAGE: Sodium Dodecyl Sulfate Polyacrylamide Gel Electrophoresis. NQ: Quantity Not Provided; NI: Not Informed; AUC: Area Under Curve; *p*: *p*-value. * Multiple epitope; ** Protein fragment; *** A full protein; **** Multiple proteins; Human: *Homo sapiens*; Capybaras: *Hydrochoerus hydrochaeris*; Horses: *Equus caballus*; Opossums: *Didelphis sp*; Pig, mice, and rabbits: species not specified.

## Pipeline for Epitope-Based Diagnostic Development

Based on a critical evaluation of case studies presented in the “Epitope Prediction and Diagnostic Application” section, we developed a modular pipeline (Figure 4) tailored for the development of serological diagnostics.

**Figure 4:**
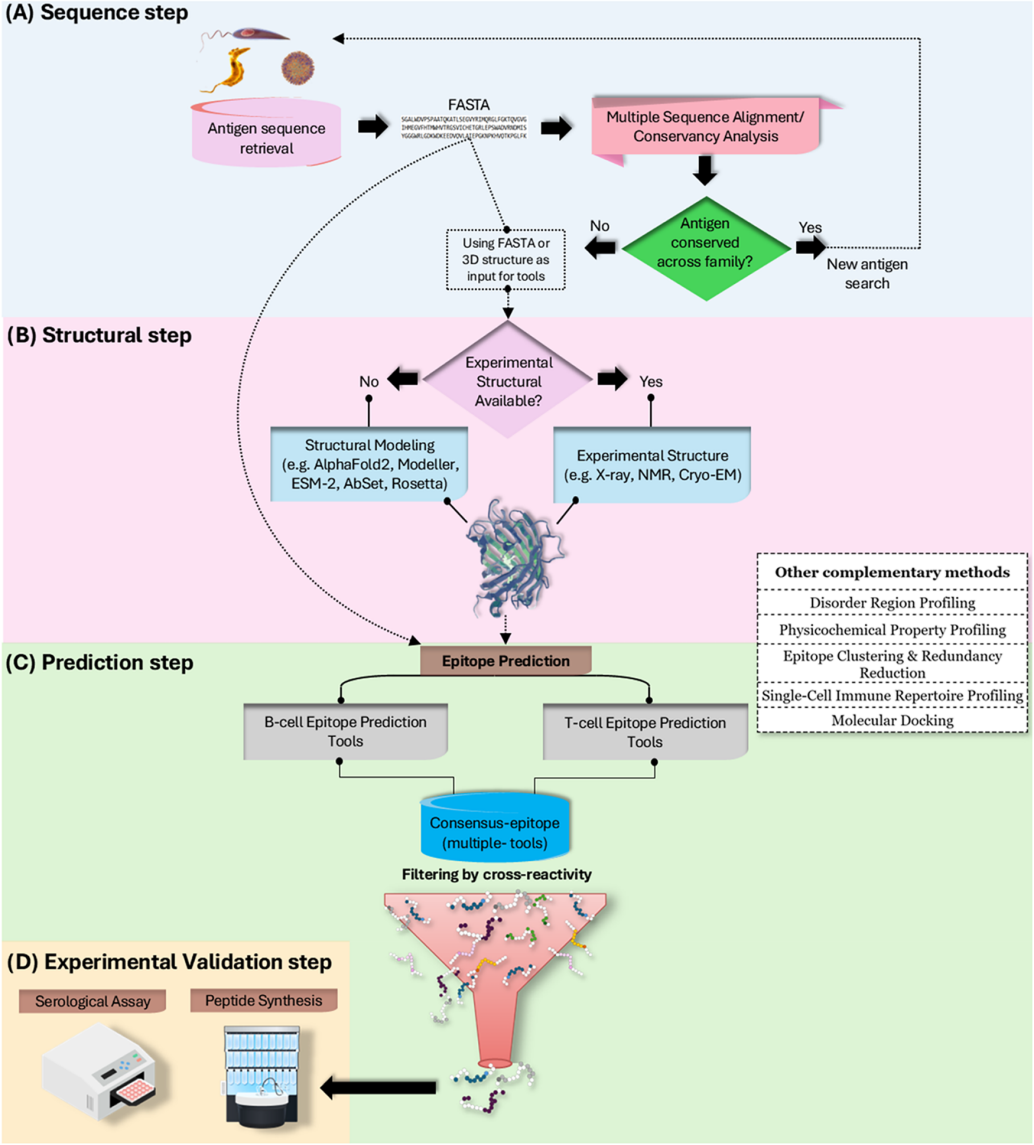
Pipeline for *in silico* diagnostic epitope selection. (A) Sequence step (light blue): selection of candidate antigens informed by pathogen biology, followed by FASTA sequence retrieval and multiple sequence alignment to assess intrafamily conservation. Antigens exhibiting high conservation among taxonomically related organisms are excluded to reduce cross-reactivity. (B) Structural step (light pink): experimentally resolved protein structures are prioritized; in their absence, high-confidence 3D models are generated. Structural data informs the prediction of B-cell conformational epitopes. (C) Prediction step (light green): B cell and T cell epitopes are predicted using complementary sequence and structure-based algorithms. Consensus regions are derived from overlapping predictions and subsequently filtered to exclude epitopes with significant similarity to host proteins or related pathogens, minimizing cross-reactivity. (D) Experimental validation step (light yellow): the top-ranked peptide candidates are synthesized and evaluated through serological assays using well-characterized positive and negative serum samples to assess diagnostic specificity and sensitivity. Icons adapted under CC-BY 4.0 from DBCLS (https://togotv.dbcls.jp/en/pics.html). The remaining graphical elements were created using PowerPoint’s native icon library.

The workflow is structured into four sequential stages (A–D), reflecting the logical progression of *in silico* epitope screening.

### (A) Sequence Step

It begins with the evaluation of the specific characteristics of the candidate antigen that influence its suitability for diagnostic applications. Next, a biologically guided preselection strategy is applied. This step also integrates the pathogen’s variability profile to support the identification of conserved regions less prone to antigenic drift. Antigens abundantly expressed during the early stages of infection are prioritized, as they improve detection sensitivity during serological windows in which antibody titers are still low.^186,187^

The cellular localization of antigen is a key factor, as proteins exposed or secreted on the surface are more accessible to the host immune system and are more likely to trigger antibody responses detectable in peripheral blood. This accessibility is crucial to the recognition process, as surface antigens constitute the primary interface between pathogens and host defenses, serving as direct targets for antibody-mediated responses.^188^

These proteins also play central roles in pathogen adhesion, immune evasion, and modulation of host responses. Studies have shown that individuals with repeated exposure to pathogens present elevated levels of immunoglobulin G (IgG) specifically directed against surface-bound antigens, reinforcing their diagnostic relevance. Consequently, such proteins are particularly valuable for the development of serological assays. In addition, prioritizing antigens with established immunogenicity, validated by experimental data demonstrating B cell or T cell reactivity, increases diagnostic robustness, reducing the probability of missed detection due to suboptimal immunological targets.^189^

Still in the sequential step, a Multiple Sequence Alignment (MSA) is performed, typically using tools such as Clustal (W or Omega),^190^ MAFFT,^191^ or MUSCLE,^192^ to compare orthologous or homologous sequences between different strains or species. To quantify this variability, Shannon Entropy is calculated at each aligned position.^193^

Highly conserved antigens across variants or related species can increase the likelihood of cross-reactive immune responses and false-positive results.^194^ To refine antigen selection and minimize this risk, the establishment of well-defined conservation thresholds becomes essential. Although some degree of sequence conservation is desirable to ensure broad detection among circulating strains, excessive conservation can compromise specificity, particularly in settings where closely related pathogens cocirculate. A balanced approach is, therefore, critical. For example, a conservation threshold of approximately 70% has been reported as a useful benchmark for selecting epitope candidates that maintain sensitivity across variants while minimizing the risk of false negatives due to sequence divergence.^195,196^ However, this threshold is not universal and may require adjustment based on specific pathogen characteristics and epidemiological context. In some cases, higher thresholds, exceeding 90%, may be necessary to avoid immune escape or ensure recognition of stable epitopes in diagnostics targeting conserved viral families.^184^

When candidate antigens do not meet the specificity or cross-reactivity criteria, the process returns to the antigen selection stage. This involves a rigorous reevaluation of potential targets, prioritizing those with distinct, immunodominant epitopes that exhibit minimal homology to host proteins or antigens from common coinfecting organisms. This iterative process enhances the likelihood of identifying diagnostically relevant targets with high specificity and translational robustness.

### (B) Structural Step

Once a suitable antigen is identified based on these criteria, its amino acid sequence in FASTA format can be submitted to various epitope prediction tools. However, for regions likely to present conformational epitopes, predictors based on 3D structural information are preferable, as they tend to increase the reliability of computational results.^73^ The pipeline distinguishes between two types of structural input. When available, experimentally resolved antigen structures, such as those deposited in the PDB, are retrieved and incorporated directly into structure-based epitope prediction workflows. Nevertheless, the scarcity of experimentally determined structures, especially for novel, emerging, or neglected pathogens, makes computational modeling a crucial alternative.^197^

Accurate three-dimensional models are generated using state-of-the-art predictive tools. Among these, AlphaFold2 ^198^ and ESM-2 ^199^ represent transformative advances in structural bioinformatics. AlphaFold2 and the evolutionary-scale prediction of protein structure at the atomic level with a language model as the ESM-2 ^199^ are transformative tools in bioinformatics, especially in structure–property–function applications, offering highly accurate structural predictions and enabling scalability and applicability of experiments. These tools have opened new avenues of investigation.

For structure-based machine learning applications, resources such as AbSet^200^ leverage comprehensive data sets of antibody structures and molecular descriptors, derived from experimentally solved and *in silico*-generated antibody–antigen complexes, systematically expanded through large-scale docking, and ranked by quality scores to enable decoy generation and high-confidence structural modeling.

Model quality is then evaluated using standardized metrics, including QMEAN.^201^ Ramachandran plots, and per-residue confidence scores (e.g., pLDDT in AlphaFold models), ensuring sufficient structural reliability for epitope mapping.^202,203^ Finally, post-translational modifications (PTMs), such as glycosylation sites, can be mapped onto the structure, as they can mask or alter antigenic determinants, impacting both epitope exposure and diagnostic specificity. This structural refinement step enhances the precision of downstream predictors, especially in identifying conformational or surface-accessible regions critical to antibody binding.

### (C) Prediction Step

Epitope predicVtion constitutes the refinement of this research, aiming to identify antigenic regions recognized by B and T lymphocytes. The accuracy and specificity of these *in silico* predictions are critical for the success of subsequent experimental validation.

In order to increase reliability, the output of multiple computational tools is integrated to derive consensus epitopes, which outperform single-tool predictions in robustness and clinical relevance. The combination of B cell and T cell predictions captures the cooperative nature of adaptive immunity, reflecting the interplay between humoral and cellular responses.^204^ As outlined in the pipeline (Figure 4), this integrative strategy mitigates tool-specific biases and leverages the complementary strengths of diverse algorithms, ultimately resulting in more accurate and biologically meaningful targets.

To enhance diagnostic specificity, predicted epitopes undergo rigorous cross-reactivity filtering based on a combination of sequence, motif, and structural criteria. This includes BLAST-based screening against the human proteome and intrafamiliar species. These steps aim to eliminate false positives by ensuring that the selected epitopes are not only immunogenic but also specific for diagnosis.

### (D) Experimental Validation Step

The epitope is chemically synthesized in this step and validated using serological assays, such as ELISA, Luminex, or peptide arrays. These assays use sera from infected and noninfected individuals to determine sensitivity and specificity under controlled conditions. This step connects computational prediction with experimental confirmation, ensuring the translatable nature of *in silico* findings to clinical diagnostics.^205^ These sequential steps constitute a modular pipeline that integrates biological relevance, structural data, and computational accuracy to support the development of serodiagnostic tools with high specificity and sensitivity.

## Prospective and Conclusion

Technological advancements have facilitated a paradigm shift in epitope prediction methodologies, with the integration of computational prediction, structural modeling, and serological validation, leading to substantial progress in identifying precise serodiagnostic markers and promising vaccines for infectious diseases. The emergence of artificial intelligence-based tools highlights the critical role of bioinformatics in epitope mapping (see Tables). However, rigorous experimental validation remains indispensable to confirm the reliability of computational predictions and refine specificity and selectivity of epitopes for future applications.

The application of multiepitope antigens has significantly improved diagnostic accuracy, particularly for pathogens with high genetic variability, where single-epitope strategies often face challenges such as cross-reactivity. To overcome these limitations, innovative strategies, such as mimotopes, extracellular vesicle proteins, and fusion antigens, which consist of multiple antigenic domains or epitopes linked in a single polypeptide to enhance immunogenicity, have been developed to enhance specificity and sensitivity in immunodiagnostics. Importantly, advances in methodologies such as AlphaFold2, ESM-2, and curated resources such as AbSet exemplify a new era in bioinformatics, where highly accurate structural predictions and large-scale, quality-controlled data sets are driving the development of robust and scalable machine learning models and expanding the frontiers of structural biology by targeting antibody–antigen complexes.

In addition to diagnostic applications, several identified epitopes demonstrate vaccine potential by effectively stimulating CD4+ T cell responses, providing an opportunity to integrate diagnostic and vaccine development efforts through targeting shared immunogenic regions. Continued advancement of computational methodologies, combined with rigorous experimental validation, will refine the identification of high-precision biomarkers for diagnostic and vaccine applications. The integration of these approaches promises to address challenges such as cross-reactivity and genetic variability, leading to more accurate and personalized strategies for managing infectious diseases. Finally, as epitope-based research evolves, its potential extends beyond diagnostics and vaccines, with the potential to reshape therapeutic approaches and contribute to global health initiatives.

## Abbreviations

ANNs: Artificial Neural Networks
APC: Antigen-presenting cell
AUC: Area under the curve
BCRs: B cell receptors
cryo-EM: cryo-electron microscopy
ELISA: Enzyme-linked immunosorbent assay
HLA: Human leukocyte antigen
IDPs: Intrinsically Disordered Proteins
IDRs: Intrinsically Disordered Regions
IEDB: Immune Epitope Database
Ig: Immunoglobulins
IMGT: ImMunoGeneTics
IUPRED: Intrinsically Disordered Prediction
LIAI: La Jolla Institute of Allergy and Immunology
mAb: monoclonal antibody
MHC: Major histocompatibility complex
NIAID: National Institute of Allergy and Infectious Diseases
NIH: National Institutes of Health
NMR: Nuclear magnetic resonance
PCR: Polymerase chain reaction
PDB: Protein Data Bank
SPR: Surface plasmon resonance
SVM: Support vector machine
TCR: T cell receptors
